# Telehealth Solutions for In-hospital Communication with Patients Under Isolation During COVID-19

**DOI:** 10.5811/westjem.2020.5.48165

**Published:** 2020-06-23

**Authors:** Jennifer Fang, Yiju T. Liu, Ernest Y. Lee, Kabir Yadav

**Affiliations:** * Harbor-UCLA Medical Center, Department of Emergency Medicine, Torrance, California; † University of California, Los Angeles, Department of Emergency Medicine, Los Angeles, California; ‡ University of California, Los Angeles, Department of Bioengineering, Los Angeles, California; § University of California, Los Angeles, Department of Medicine, Division of Dermatology, Los Angeles, California; ¶ David Geffen School of Medicine at UCLA, UCLA-Caltech Medical Scientist Training Program, Los Angeles, California; || The Lundquist Institute for Biomedical Innovation, Torrance, California

## Abstract

The coronavirus disease 2019 (COVID-19) pandemic is a public health crisis that has quickly overwhelmed our healthcare system. It has led to significant shortages in personal protective equipment (PPE), ventilators, and intensive care unit beds across the nation. As the initial entry point for patients with suspected COVID illness, emergency departments (ED) have had to adapt quickly to prioritize the safety of patients and providers while still delivering optimal, timely patient care. COVID-19 has presented many challenges for the ED that also extend to all inpatient services. Some of these key challenges are the fundamental tasks of communicating with patients in respiratory isolation while minimizing PPE usage and enabling all patients who have been affected by hospitals’ visitor restrictions to connect with their families. We discuss the design principles behind implementing a robust in-hospital telehealth system for patient-provider and patient-family communication, provide a review of the strengths and weaknesses of potential videoconferencing options, and deliver concise, step-by-step guides for setting up a secure, low-cost, user-friendly solution that can be rapidly deployed.

## INTRODUCTION

We have taken for granted the convenience of evaluating our patients directly by walking into rooms and having a conversation. The traditional workflow of taking a history, performing a physical exam and diagnostic tests, monitoring treatments, and disposition planning necessitates multiple bedside interactions between patients and hospital staff. The coronavirus disease 2019 (COVID-19) pandemic has caused a national shortage of personal protective equipment (PPE).^1^ With so many in respiratory isolation and a limited supply of PPE, how do we adapt patient-provider communication to minimize unnecessary entry-exit cycles?^2^–^4^ Additionally, hospitals’ bans on visitors have impacted all patients, not only those with COVID-19.^5^,^6^ Hospitals worldwide are seeking methods to communicate with patients under isolation precautions while protecting their staff, efficiently using PPE, and enabling patients to virtually be with their families at a time when they are ill and alone.^7^,^8^ Given the current crisis, rapid deployment of communication solutions is urgently needed.^3^,^7^,^9^ Some hospitals are already integrating telehealth into their workflows, but many are unsure how to do so appropriately.^10^–^12^

Prior to this pandemic, only Health Insurance Portability and Accountability Act (HIPAA)-compliant software offering business associate agreements (BAA) were allowed for medical use. Even if the same company offered a free version of the same software, it could not be used because no BAA agreement existed between the company and the hospital. However, with the sudden demand for accessible telehealth options, the Department of Health and Human Services (HHS) temporarily expanded allowable applications (apps) and “will not impose penalties for noncompliance with the regulatory requirements under the HIPAA Rules against covered healthcare providers in connection with the good faith provision of telehealth during the COVID-19 nationwide public health emergency...us[ing] any non-public facing remote communication product that is available to communicate with patients.”^13^

Current telemedicine technologies leverage mobile devices and high-speed Internet access to connect patients with providers. Numerous established companies and startups offer telemedicine products. Typically, telemedicine platforms are used for consultations^14^–^16^ or remote treatment monitoring.^17^–^20^ Few studies have focused on implementing real-time videoconferencing in the emergency department or inpatient settings,^16^,^21^ and none have described a specific implementation strategy for doing so within the unique constraints of the COVID-19 pandemic.

Our academic medical center is part of the second-largest public hospital system in the country, and, like many, we are challenged with limited PPE stock and readily accessible funding for telehealth equipment and software. To address this unmet need for in-hospital patient communication, we developed a cost-effective plan for rapid implementation with minimal equipment and setup. In this article, we discuss the design principles to effectively implement such a solution and compare common videoconferencing apps. Based on these factors, we produced a step-by-step guide to implement the protocol we deployed in our health system (see Supplements 2–3 for detailed guides). We hope that this work provides a blueprint for how resource-limited hospitals can rapidly implement an affordable, in-hospital telehealth communication solution during the COVID-19 pandemic.

## GOALS

### Staff Communication

With limited PPE supply and exposure risks associated with frequent PPE doffing, hospitals should minimize entry-exit cycles by necessary staff to isolated patients’ rooms.^10^,^22^–^24^ Staff who only need to speak to the patient should not have to enter the room at all.^9^ Examples include registration clerks, case managers, and social workers. Room entry is not necessary for answering many patient questions, updating care plans, or recording during a resuscitation. In the case of teaching hospitals, the entire team does not need to enter the room; attendings and other learners may stand outside to observe the patient encounter.

### Family Communication

A vital part of humanistic care for all patients during this crisis is to provide a way for them to connect with their families. When patients are under isolation precautions, visitation is restricted.^25^ During the current pandemic, some hospitals have instituted blanket “no visitors” policies^5^ for all patients, which can have significant detrimental impacts on patient mental health and recovery.^26^–^28^ Both COVID-19 and non-COVID-19 patients admitted to the hospital are often quite ill, feel isolated from their loved ones,^29^ and may be faced with daunting goals of care conversations.^30^ For patients who do not speak English, do not own a mobile phone, or are not used to navigating the healthcare system alone, isolation creates additional anxiety.^31^

## DESIGN PRINCIPLES FOR COMMUNICATION SOLUTIONS

With the above goals in mind, we factored in cost of implementation, privacy concerns,^32^ administrative overhead, and ease of use in practice into the final design choices. In addition to hospital-provided videoconferencing solutions to the communication problem, multiple low-tech methods were considered (Supplement 1). We provide an analysis of the advantages and disadvantages of each method.

Major advantages of video communication are that it provides a more personal connection and participants can better assess non-verbal cues. Implementation is limited by high upfront costs of purchasing devices and HIPAA-compliant software for videoconferencing. Without hospital-backed funding, the recurring costs of these subscription services become prohibitive. However, during the COVID-19 crisis, the most recent HHS notice^13^ enables providers who are otherwise unable to afford HIPAA-compliant technologies to leverage free software that meets these requirements to provide urgent patient care.

### Device Costs

The upfront cost of buying tablets may be restrictive for resource-limited hospitals. The most common tablets run one of three operating systems (Android, Windows, iOS), and cost approximately $50–$500. If the hospital cannot buy particular devices because of funding or contractual constraints, community donations of used tablets are another option.

### Device Security

To restrict user access to other applications and device settings, tablets may be placed in “kiosk mode,” a feature commonly used in retail that is available on Android (screen pinning), Microsoft Surface (kiosk mode), and iOS devices (guided access). All three major platforms also offer enterprise management solutions to set up and electronically secure devices. The limitation of Microsoft Surfaces is that, other than Skype, most conferencing services are not native apps available through the Microsoft Store and may have to be used in the web browser; thus, browser restrictions would also need to be set.

### Patient Privacy

Although HHS will not penalize hospitals for using software that is not officially HIPAA compliant during the COVID-19 pandemic, hospitals must still ensure patient privacy when implementing telehealth solutions. Depending on the chosen app, the methods to maintain patient privacy are either to create unique accounts for each patient or to choose an app that only allows calls from an approved contact list. If calls cannot be restricted to a given list, there is a risk of strangers calling patients.

As the devices will be used with multiple patients, video capture and screenshots should be disabled so that recordings or photos of staff or patients are not stored on the device. With standard, off-the-shelf devices rather than enterprise devices, it is not possible to globally disable device screenshot settings, but individual apps may restrict screenshots. For apps that do allow recording or screen capture, hospital staff would need to verify that everything is deleted from the device after each use.

### Staff Safety

By using commonly available free apps for patient communication, hospital staff may wish to use their own devices for expediency. However, this access should be prohibited for both patient privacy and staff safety. Staff members’ personal accounts should not be able to call hospital devices, and patients should not have access to staff members’ personal contact information. Instead, there should be additional hospital accounts or devices available for staff to call patients. The device and app settings must also be configured so that patient-facing devices are secure from settings changes and unapproved downloads.

### Usability

User-friendly apps decrease the need for staff to repeatedly enter patient rooms to help patients use the devices, which would negate efforts to limit exposure and PPE usage. Apps should be easy to use and have limited menu options; multiple menus are confusing and make initiating calls difficult.^33^ For programs that are only available via a web browser (rather than a native app), patients could accidentally close the tab and have difficulty returning to the app without staff assistance. All common operating systems also include accessibility settings, which enable larger font sizes for patients with decreased vision. When possible, these should be enabled by default.

Although staff can help a patient troubleshoot the app, if the family is not familiar with the corresponding app, hospital staff will have difficulty remotely helping the family troubleshoot. Apps that generate a website link, instead of requiring family to download an app or create an account, will be the most broadly accessible. Of the apps tested, only Zoom provides this option.

### Administrative Overhead

Unlike with HIPAA-compliant enterprise versions, free services have less granular control over app settings. For apps where settings are accessible by patients from within the app, these settings have to be re-verified between patients.

Videoconferencing apps enable patients to see and speak to family members who are not allowed to visit. Adding family members’ contact information to an app creates minimal administrative burden while bringing great psychological and emotional benefit to patients. However, giving family members account information to reach their loved ones also means giving families future access to other patients if settings are not configured properly.

Apps must have settings that restrict contacts and maintain anonymity, or unique accounts must be created for each patient in order for shared devices to maintain patient privacy. For apps with settings that restrict calls to approved contacts only, new accounts do not need to be generated between each patient use; however, call and chat logs should be deleted so that the next patient cannot see prior conversations or non-hospital contacts. Enterprise management solutions offer remote device resets between patients, but may not be able to remotely clear the call and chat logs of individual apps. For apps that do not restrict callers, the administrative burden of generating unique accounts for each patient or even asking patients to create their own accounts is high.

Free Google accounts are limited to 10 lifetime accounts per person; thus, non-enterprise creation of free unique accounts for Google-based apps is not sustainable. Regardless of which devices or apps are used, at minimum, accounts will need to be created for the devices. For those who are unable to provision enterprise accounts, for non-Google products a domain name can be purchased for approximately $10 per year and used to generate an unlimited number of usernames that route to a single email account for easy account and password management.

## COMPARISON OF FREE APPLICATIONS

We compared the advantages and disadvantages of four well-known, commonly available free videoconferencing apps (Table). The app features described in the table address the principles of security (app settings hidden from patients, encryption); patient privacy (calls restricted to contacts only); usability (cross-platform, dials landlines); and administrative overhead (call logs). Another major usability factor is the user interface (UI). FaceTime and Google Duo have simple UIs where the focus of the app is to make a call. The other apps have multiple tabs for chats, calls, contacts, or settings.

## DISCUSSION

Ultimately, based on ease of setup, patient privacy settings, UI simplicity, and ease of between-patient maintenance, we implemented our protocol using FaceTime. Choosing FaceTime limited us to using iPads rather than Android devices, which can cost less. However, by repurposing existing devices and using donated devices that our health system received from a nonprofit organization, our total device and application cost was $0.

Compared to other options, FaceTime was the easiest to set up. FaceTime comes preinstalled. Other than each device’s login information, no additional downloads or accounts needed to be made. FaceTime is the only free app we tested that fully hides app and device settings from patients when both kiosk mode and parental controls are activated. These restrictions and the absence of chats allow for the greatest device security, patient privacy, and ease of between-patient maintenance. Unlike Zoom, Skype, or Google Hangouts, FaceTime has only one function: making calls. FaceTime does not have additional menus that patients, particularly non-English-speaking patients, could be confused by, thereby decreasing provider time required to teach patients how to use the app.

Although FaceTime has superior usability and security advantages, the major drawback is that FaceTime is only available on Apple products. This limitation does not affect in-hospital communication with staff, but patients can only call loved ones with Apple devices. To enable patients whose families do not have Apple devices to make calls, we set up an on-site family call center. Regardless of the app chosen, using apps to call families will create barriers for those who do not have tablets or laptops, have difficulty downloading apps or setting up accounts, or have limited access to the Internet. Offering a call center where families can use hospital-provided tablets would address this limitation.

We worked with multiple stakeholders—including patients, staff, hospital administration, clinical informaticists, infection control, and facilities management—to implement the optimal solution for our health system. Engaging hospital and health system leadership early enabled us to seek approval from the various branches in parallel, expediting the process. Enterprise solutions are preferred for easy, standardized maintenance, but can be cost-prohibitive.

In recent weeks, hospitals have attempted to rapidly expand in-hospital telehealth, and preliminary experiences have been positive.^34^,^35^ Whereas most pre-pandemic telehealth tools targeted outpatient care, the increased demand for in-hospital usage creates opportunities for new solutions. Now that we have implemented a telehealth solution in our hospital to address this care gap, we plan to conduct a longitudinal study to quantify the value of these tools to patients and providers in facilitating communication and improving quality of care. The success of programs like ours would provide justification for health systems to invest in HIPAA-compliant solutions post-pandemic or regulatory bodies to expand the definition of HIPAA-compliant software. Moving forward, in-hospital video telemedicine use can be expanded beyond communicating with isolated patients to enhance the following processes: increased ability of offsite consultants to perform limited evaluations; safer triage practices during future pandemics, and minimizing staff during resuscitations by enabling additional staff to safely observe from outside the room.

Our in-depth analysis presented here can guide readers seeking to expand in-hospital telehealth capabilities by adapting existing systems based on these design principles for their own hospitals. For readers in need of an immediate solution during this pandemic, we provide detailed, step-by-step setup and usage guides (Supplements 2–3) for the solution we implemented. We believe that our novel work will serve as a blueprint for how resource-limited hospital systems can quickly implement a secure, low-cost, user-friendly telehealth communication solution to safely care for a large number of isolated patients while conserving PPE usage during the ongoing COVID-19 pandemic.

## Supplementary Information







## Figures and Tables

**Table:** Videoconferencing applications and features.

Service	Cross-platform^a^	App settings hidden^b^	Restricted contacts^c^	Dials landlines^d^	E2EE^e^	Call logs only^f^	Additional factors
FaceTime	No	Yes	Yes	With cell provider	Yes	Yes	--
Google Duo	Yes	No	No	No	Yes	Yes	Free version limit of 10 lifetime accounts per person
Google Hangouts	Yes	No	Yes	Yes	No	No	Free version limit of 10 lifetime accounts per person
Skype	Yes	No	Yes	$3/account/month for unlimited calls within United States	Option*	No	On iPhone only, unable to disable integrated calling, so both app and device call log need to be cleared
Zoom	Yes	No	Yes	Price varies based on usage	No	No	

*E2EE*, end-to-end encryption; *app*, application.

* Skype provides an E2EE option for chats and audio calls, but the option is difficult to find and must be reselected via multiple menus each time you initiate a call.

a Cross-Platform: Available on multiple different operating systems and devices.

b App Settings Hidden: If the app settings are visible and editable by patients, the settings would need to be manually checked and reset after each use.

c Restricted Contacts: If an app is unable to restrict calls to contacts only, in order to maintain patient privacy a new account or password would need to be generated for each patient. Even if an app can restrict contacts, if the app settings aren’t hidden, the patient may still be able to remove the restriction within the app. The only free service that we tested that provides full restriction is FaceTime.

d Dials Landlines: Services that do not offer free calls to landlines limit the ability to call a translator or loved ones without smartphones or computers. Services that require an associated cell phone number cost upwards of $15 per month for unlimited calls.

e E2EE: All of these services offer some degree of encryption. E2EE is the most secure form of encryption; only the people in the conversation can see or hear messages; no third parties can decrypt any transmitted data—even the company that makes the product.

f Call Logs Only: Apps that enable typed chats generate chat logs, which, in addition to call logs and contact lists, need to be deleted after each patient’s use.
