# Supplementary material for: Telehealth Solutions for In-hospital Communication with Patients Under Isolation During COVID-19
Source: West J Emerg Med. 2020 Jun 23;21(4):801–6. doi: 10.5811/westjem.2020.5.48165 (PMC7390554; doi:10.5811/westjem.2020.5.48165)
Supplement: Supplementary file 1 [file wjem-21-801-s001.docx]

**Supplement 1.** Discussion of communication options for patients under isolation.

*Low-Tech Solutions*

Whiteboards or pieces of paper used for written communication restrict usage to patients who are literate and have good vision. Written communication is further limited by poor handwriting and does not allow use of a certified translator if one is not physically present in the hospital. Using the patient call-button speaker system may work for some hospitals; our hospital’s patient call buttons vary in quality throughout the hospital; so this was not a reliable method of communication. Walkie talkies have better sound quality, but using walkie talkies has usability challenges. Patients may accidentally change the radio channel, which allows them to talk to each other or listen in on other provider-patient discussions. This problem requires a provider to physically enter the room to troubleshoot. Baby monitors were considered due to their affordability, but they have limited range, poor sound quality, and are always on, risking noise and alarm fatigue when clustered at a nursing station (Table S1). None of these low-tech solutions allow patients to communicate with loved ones unless they are physically in the hospital, and visitors are heavily restricted during the pandemic.

*Higher-Tech Solutions*

Portable hospital phones are a great option if they are available, but not all of these phones can call outside the hospital network. During this international emergency, our hospital has also encountered difficulty in obtaining more of these portable phones for patient use. Further, hospital phones don’t support videoconferencing, which would be preferable for communication with providers and offsite loved ones.

Allowing patients to use their personal phones^1,2^ maximizes the number of devices available for communication at no additional cost to the hospital.^3^ However, calls from patient devices are limited by in-hospital cellular network reception, a problem in many hospitals as a result of building construction materials, and availability of appropriate chargers if patients do not have their own. Furthermore, allowing patients to call the nursing station directly dilutes other important calls made to hospital phones (Table S1). The increase in untriaged calls contributes to work distractions and missed provider calls, worsening overall communication within the hospital. To avoid giving out the direct unit phone numbers, HIPAA-compliant telehealth applications such as Zoom for Healthcare, Skype for Business, and Google’s G Suite can be downloaded onto patients’ devices with their consent. This method requires patients to have compatible devices, potentially uses patients’ cellular data plans, and imposes an unneeded learning curve during an already stressful time. Providing technical support for patients to set up their own phones creates additional provider burden, which is exacerbated by the sheer diversity of mobile devices and language choices.

| Method | Advantages | Disadvantages |
| --- | --- | --- |
| Whiteboards or paper | - Easily accessible - Comparatively inexpensive - Easy to decontaminate or dispose of securely - No logs to clear | - Patient must be literate - Unable to use with patients with vision problems - Limited by poor handwriting - If no in-hospital translator, cannot be used with non-English speaking patients - Both parties must be physically present - No video communication |
| Patient call button | - Built into hospital rooms already - Familiar to patients and staff - No logs to clear | - Poor sound quality - Poor interface with translator phone - Both parties must be physically present - No video communication |
| Walkie talkie | - Easy to set up - Lower cost than most technology solutions - No logs to clear | - Potential for patients to listen to other patient-provider conversations or talk to each other - Accidental channel changes result in staff needing to go into room to reconnect patient - Poor interface with translator phone - Limited range - No video communication |
| Baby monitors | - Easy to set up - Lower cost than most technology solutions - Can provide both audio and video communication - No logs to clear | - Poor interface with translator phone - For low-cost versions without an associated app, there is limited range (mostly line-of-sight) - Always on, which can generate constant noise if multiple monitors at nurses station |
| Portable hospital phones or VoIP phones | - Existing technology in most healthcare settings | - May not allow calls outside of hospital network to patients’ families - Additional units expensive - Easily misplaced - No video communication |
| Personal phones | - Patients are familiar with their own phones - Allows communication with loved ones offsite - Increases the number of available devices - No cost to the hospital - No logs to clear | - Limited by in-hospital cellular network and Wi-Fi reception - Using personal phone to call the unit phone increases call volumes - If app installation is required, limited by patient’s technology literacy - May not support video communication |
| Tablets | - Provide high-quality audio and video communication - Familiar to patients who have tablets at home - Allows communication with loved ones off site | - High upfront cost unless donated - Usage of video or calling applications typically restricted to HIPAA-compliant software - May need to clear call and/or chat logs |

**Table S1.** Advantages and disadvantages of communication methods.

*app*, application; *VoIP*, voice over Internet Protocol; *HIPAA*, Health Insurance Portability and Accountability Act.

**REFERENCES**

1. Arora S, Burner E, Terp S, et al. Improving attendance at post-emergency department follow-up via automated text message appointment reminders: a randomized controlled trial. *Acad Emerg Med.* 2015;22(1):31–7.
2. Arora S, Peters AL, Burner E, et al. Trial to examine text message-based mHealth in emergency department patients with diabetes (TExT-MED): a randomized controlled trial. *Ann Emerg Med.* 2014;63(6):745–54.e6.
3. Arora S, Ford K, Terp S, et al. Describing the evolution of mobile technology usage for Latino patients and comparing findings to national mHealth estimates. *J Am Med Inform Assoc.* 2016;23(5):979–83.
