# Supplementary material for: Telehealth Solutions for In-hospital Communication with Patients Under Isolation During COVID-19
Source: West J Emerg Med. 2020 Jun 23;21(4):801–6. doi: 10.5811/westjem.2020.5.48165 (PMC7390554; doi:10.5811/westjem.2020.5.48165)
Supplement: Supplementary file 2 [file wjem-21-801-s002.docx]

**Supplement 2.** iPad setup instructions.

[**Goal**](#_kcejsqf5bz65) **1**

[**System Overview**](#_swjzsadza410) **2**

[Hardware](#_21bardnkw01f) 2

[Software](#_w98efns3whzk) 2

[Setup](#_un676gjexl2i) 2

[Apple Configurator 2](#_orwn4flmy168) 2

[Manual Setup](#_ij0yen47qn6g) 2

[Security and Patient Privacy](#_bfenjurgdx9i) 3

[Passcodes](#_jhtt2oxcd6rk) 3

[iOS Security and Privacy Features](#_o2dz3rpjkc0j) 3

[**Accounts**](#_6fjntq5lx7mj) **4**

[Creating Apple Accounts](#_i1kjulfn4gma) 4

[Family Sharing Accounts](#_641oztkj9tg4) 5

[Advantages](#_lgdcdpnuyd2h) 5

[Disadvantages](#_il8i9q5cg01e) 6

[Making Accounts](#_9y41tm22z5tf) 6

[**iPad Setup**](#_4zg9ywc4woqp) **7**

[Apple Configurator 2 (AC2) Setup](#_duq6bg8nizkv) 7

[Set Up Apple Configurator 2](#_lrn9s7g7git4) 7

[Set Up iPads](#_2ab8uaieec6m) 9

[Manual Setup](#_acpht4w5zjwp) 10

[**iPad Preparation for Use**](#_gxxukusiaj6) **13**

# Goal

This document will cover the system for establishing in-hospital communication between patients and hospital staff or patients and their families. The system securely uses iPad devices and no additional software. The system centers around using FaceTime and Apple’s built-in device restrictions to ensure that patient privacy is maximized while giving hospital staff access to patients virtually.

This document will generally use the term iPad but the system will work on any Apple device running iOS 12 or later (iPod, iPhone, etc.)

# System Overview

## Hardware

The system will consist of:

1. “Hub” or “Staff” iPads. These iPads are to be used by the hospital staff to communicate with patients.
2. “Patient” iPads. These are the iPads that will be given to patients for patients to call the hubs or their families.

## Software

On the iPads, we use Facetime, Apple’s secure peer-to-peer communication app. Facetime uses 256-bit AES encrypted audio/video between iPads. The encryption is performed on a per-session basis.

## Setup

The setup process for the iPads can be done either manually or via Apple Configurator 2 (preferred).

### Apple Configurator 2

Apple Configurator 2 (AC2) is an enterprise management solution that can be used to quickly and easily configure a large volume of iPads. To use AC2, you will need a Mac that runs MacOS 10.14.6 or later. If you have access to a computer meeting these requirements, we highly recommend that you use AC2 instead of performing manual setup because AC2 decreases setup time, reduces likelihood of setup mistakes, and provides greater security restrictions on the device.

AC2 also allows you to lock an iPad to a single app and thereby not need to use Guided Access mode which simplifies the system. When locking the iPad to the app you can also turn off the ability for the user to use any of the buttons in the system, rotate the screen, etc.

### Manual Setup

If you do not have access to Apple Configurator 2, manual setup is easy and secure. All setup can be done directly on the iPad. No Mac is needed for setup.

## Security and Patient Privacy

### Passcodes

Depending on the setup method used, not all passcodes may be used. Which passcodes hospital staff have access to also depends on the setup method. There are up to four security measures in places on the devices:

1. Apple ID Password: protects Apple account that the iPad is logged into
2. Device Passcode: entered on lock screen to unlock iPad and for certain device settings
3. Screen Time Passcode: used to edit “parental control” restricted settings such as contacts
4. Guided Access Passcode: used to exit the one app that the iPad is locked into in Guided Access mode. In this case, it will be used to exit FaceTime

### iOS Security and Privacy Features

In addition to standard device passwords, iOS 12 and later come with “Screen Time,” settings designed for parental controls, which provides additional security features that allow us to ensure patient privacy.

1. Apple Accounts: Each iPad will have its own Apple account.
2. Device Passcode: The iPad will have a 6-digit passcode to unlock the iPad. The passcode is required when the iPad is restarted or the screen is locked.
3. Screen Time
   1. Using Screen Time, we can enable to following security features (iii-v important if the patient iPad is not restricted to FaceTime access only, either by choice or by forgetting to re-enable Guided Access mode)
      1. Restrict FaceTime Calls to Contacts Only
         1. This feature restricts incoming and outgoing FaceTime calls for this iPad to contacts only, so strangers cannot call the iPad
      2. Restrict ability to edit contacts
         1. The user of the iPad cannot edit the contacts list.
      3. Restrict ability to use certain apps
         1. All apps except for FaceTime and Camera (required for FaceTime) are restricted. Using the Messages app will not be allowed.
      4. Restrict ability to install other apps
      5. Restrict ability to change certain iPad settings
   2. Screen Time can be set up so that the above settings are always on
   3. Screen Time enforces these restrictions through a 4-digit Screen Time passcode
4. Guided Access Passcode
   1. “Guided Access” mode, Apple’s version of a kiosk mode, restricts the iPad to a single application. It requires a separate 6-digit passcode (different from device passcode) to resume normal iPad use and switch applications. With Guided Access mode on, the user will only be able to use the FaceTime app.
5. Additional security settings such as turning off backups to the cloud will be set in the “iPads Setup” section below

# Accounts

## Creating Apple Accounts

A surprisingly tricky aspect of the system is how to generate many Apple accounts. Each Apple account requires a valid email address through an external service such as Gmail, Outlook, etc. Each iPad needs its own apple account for the following reasons:

1. An iPad’s Apple account email address is used to initiate a FaceTime call. If multiple iPads are logged into the same account, they will both ring. This behavior may be desired for the hub iPads but not for the patient iPads.
2. Each Apple account can sync its contacts from device to device. Since we are relying on Screen Time’s ability to only let FaceTime communicate with contacts, having separate contact lists preserves patient privacy.
3. Apple also has the ability to sync other account data across devices. There is a risk of an unexpected data breach between patients if patient iPads share accounts.

Given that dozens of accounts may be needed, we need to be able to easily, rapidly, and cheaply generate valid email addresses that can comply with email account creation rules and receive account verification emails from Apple. Apple’s account creation rules are not well documented, but based on our testing, the following methods do not work:

1. Creating emails with an existing email provider (Gmail, Outlook, Yahoo! Mail, etc.) can be labor and time intensive. Some popular email providers, such as Gmail and Outlook, limit the number of free, basic accounts that can be generated per person per lifetime (based on a required cell phone number tied to the accounts), so we could not create a large number of Apple accounts with this method.
2. Using Gmail, in some websites, appending a “+” and another word to the base email username registers as a separate account while still sending all messages to the base email. For example, if an account is created with “emailname@gmail.com” some websites will accept “emailname+1@gmail.com” as a new account name while still sending all messages to “emailname@gmail.com”. Apple recognizes emails with “+” as the same account.
3. Using an email domain forwarding service, such as 33mail, to create an infinite number of emails is also blocked by Apple. For example, if one purchases the domain “name.33mail.com” for $12/yr on 33mail and then attempts to create an account with “account@name.33mail.com”, account creation fails with an unidentified error when attempting to enter the 6-digit verification code.

If your hospital is able to provision enterprise accounts, the IT team can generate new accounts for you. If you are unable to use enterprise accounts, a fast, inexpensive solution is to use a Domain Name Registrar to purchase a new domain (we purchased one for $12 during testing). The domain can be set up so that all emails sent to the domain are forwarded to a single email. Different registrars will make this purchase and configuration complex/easier than others. The service we used, Namecheap, was simple to set up. Within 10 minutes (Namecheap claims it may take up to 60), emails sent to any email address at our domain (such as test@domain.net or staff@domain.net) were being forwarded to the single email address we are using for this project. We used Gmail for our master account, but this method will work with any email provider. With this solution, no additional email accounts need to be made.

With the domain in place, Apple IDs can easily be created. Based on our testing, Apple does not seem to rate limit account creation when it is done manually, even from a single computer.

1. Go to [appleid.apple.com](http://appleid.apple.com) and click “Create your Apple ID” button in the upper right portion of the page
2. Fill out the account information and save the information in a secure file for your administrators.
   1. Apple IDs will be in the username@domain.net format.
   2. Create a unique password for each Apple ID.
3. Open your master account to get the verification code and enter it on the website to complete account creation.

## Family Sharing Accounts

If you have enough iPads to make more hubs and support a 1:5 hub:patient iPad ratio (rather than 1 hub to 6+ patients), we recommend using the Family Sharing modification because it makes it easier for staff to add/remove contacts from the patient iPads.

Apple Family Sharing, a parental control setting designed to allow parents to remotely control their children’s iPads, allows a maximum of five “child” (patient) accounts for each “parent” (hub), and together the hub and 5 “child” iPads are one “hub family.”

### Advantages

With the Family Sharing method, staff can remotely add/remove contacts from a patient iPad rather than needing to go into the room to make direct changes to the patient iPad. This method removes the need for staff to use passcodes or access multiple apps on the patient iPad when resetting it.

Furthermore, because addition and deletion of contacts can be done remotely, if a patient wants to add another contact, the patient can call the hub, tell staff the phone number, and staff can add this contact to the patient’s iPad immediately.

### Disadvantages

A disadvantage of Family Sharing is that “child” (patient) Apple accounts must be created from the Hub iPad before the patient iPads can be set up. In order to be associated with the correct hub, the patient iPad accounts cannot be created in advance.

Another disadvantage for Family Sharing is the need to add payment information (credit card or PayPal) to your hubs’ Apple accounts in order to set up each “family.” Using the settings described later in this document, we do not anticipate any charges being made to the payment method based on the secure iPad setup. However, to lower the risk of charging a hospital or personal payment method unnecessarily, we recommend entering the information for a low-cost, low-balance prepaid credit card.

### Making Accounts

If using Family Sharing to make accounts, make the following modifications to the account setup process. These steps must be done from the Hub iPads.

1. Use the “Creating Apple Accounts” protocol to make Hub accounts
2. Proceed through setup of a Hub device (see setup methods in the “iPad Setup” section of this document)
3. Once the Hub device is set up, open the Settings app.
4. Click on the Apple ID left-side menu just below the search bar and an account menu will appear on the right side of the screen
5. Click “Payment & Shipping” to add a payment method. A payment method must be on file in order to use Family Sharing but should not get charged.
6. Click “Set Up Family Sharing.” In the popup window, click Get Started > Screen Time > Create a Child Account > Next and complete the prompts to add the first child. You will only be able to make an “@icloud.com” email address.
7. From the “Family Sharing” menu, repeat the following steps to add additional “child” or patient iPad accounts that will be associated with this Hub.
   1. Click Add Family Member > Create a Child Account > Click “Next” when the popup appears and complete the prompts. You will only be able to make an “@icloud.com” email address.
   2. Click through the rest of the prompts until you get to a “Screen Time” prompt. Click “Continue”
   3. Click “Not Now” for Downtime
   4. Click “Not Now” for App Limits
   5. Click “Continue” for Content & Privacy
   6. Set a Screen Time passcode for the patient iPad
   7. Click Done
8. Go back to the iPad Settings > Screen Time (located on the left side menu) to set up the contacts-only call restriction on each patient iPad. Repeat the following steps for each patient iPad account.
   1. Under the “Family” section, click on the patient iPad’s name. If you see “Turn On Screen Time,” click it and set a **Screen Time Passcode**.
   2. Click “Communication Limits”
   3. Under the “Allowed Communication” header, click “During Screen Time” > “Contacts Only”
   4. Turn on “Manage Contacts.” This setting allows the Hub iPad to remotely add/remove approved contacts for each patient iPad
   5. Do not add contacts to the patient account at this time (before the patient iPad has been set up below) because contacts will not appear unless added after the patient iPad has been set up.
   6. Turn off “Allow Contact Editing”

# iPad Setup

iPad setup varies depending on whether manual setup or AC2 is used. AC2 is recommended. See the “Setup” section under “System Overview” for a comparison.

## Apple Configurator 2 (AC2) Setup

### Set Up Apple Configurator 2

First, you will need a Mac that runs macOS 10.14.6 or later. Install Apple Configurator 2 from the App Store. When setting up AC2, at some point during setup you will be prompted for an Apple ID. All iPads set up with AC2 will belong to this Apple ID instead of the Apple ID of the individual iPad accounts.

**Create An Organization**

After installing AC2, create a new organization to which all the iPads will belong (for example, your hospital name).

The steps to create an Organization are below:

1. Click on “Apple Configurator 2” in the menu bar, then click on “Preferences.” A popup menu will appear.
2. Click on the “Organizations” Tab
3. In the bottom left corner of the popup menu, click the “+” button to “Create an Organization”
4. Click “Next.” When prompted for an Apple ID, click “Skip” in the bottom right corner.
5. Enter organization contact information and click “Next”
6. Choose “Generate a new supervision identity” and click “Done”
7. Close the Preferences window.

**Create Blueprints**

Blueprints are the configurations that specify how to set up a device, including iPad settings, which apps will show up on the iPads, and restricting single-app access. Create one blueprint for the patient iPads and one blueprint for the hub iPads. The following steps are performed in Apple Configurator 2.

1. On the menu bar, click “File” > “New Blueprint.” A new Blueprint will appear on the screen with the name “Untitled” highlighted and ready for renaming.
2. Name the blueprint, such as “Patient” or “Hub”
3. Double-click on the blue box/blueprint icon to open the blueprint
4. If you have already created a profile, skip to step 5. If you have not yet created a profile, on the menu bar, click “File” > “New Profile” (a profile is a group of settings). To edit an existing profile if you have made one previously, click “File” > “Open” and select your profile file. A new window will open with all possible profile settings. Multiple settings may be preset, but this document will only cover the minimum necessary for our patient communication goals.
   1. Choose “Restrictions” from the left side menu. By deselecting most of the options in these menus, we have disabled screen shots, iCloud photos, purchases, app installation, etc.
      1. From the first tab, the “Functionality” tab, uncheck all options except the following 5 items
         1. “Allow use of camera” and “Allow FaceTime”
         2. “Allow iCloud documents & data” and “Allow managed apps to store data in iCloud” (necessary if remotely managing patient iPads’ contacts from the Hub iPad)
         3. “Allow Screen Time” (necessary to restrict calls to contacts only)
      2. From the second tab, the “Apps” tab, uncheck all options.
         1. Under “Restrict App Usage,” select “Only allow some apps”
         2. Click the “+” to add the following apps.
            1. Contacts
            2. FaceTime
            3. Settings
   2. Close the profile editing window and click “Save” when prompted
5. Load the profile into the blueprint by clicking “Actions” (in menu bar) > “Add” > “Profiles”
6. On the menu bar, click on Actions > Modify > Home Screen Layout. Depending how you plan to set up the patient iPads, this step may only apply to the Hub iPads.
   1. Modify the layout so that the following apps are in the home dock part of the layout
      1. Contacts
      2. FaceTime
      3. Settings
   2. The remaining apps can be left where they are because they will be disabled

Family Sharing Accounts

If you are using Family Sharing accounts (see “Accounts” section of this document), for the patient iPad blueprint, perform these additional steps to restrict the iPad to FaceTime only. These settings will eliminate the need to use Guided Access mode.

1. On the menu bar, click Actions > Advanced > Start Single App Mode. A window will pop up.
2. Click the FaceTime icon
3. Click “Options” in the button in the bottom left corner. Another window will pop up.
4. If you want to disable the physical iPad buttons, leave only “Touch” and “Motion (Screen Rotation)” checked. The “Touch” option is necessary to make and answer calls from FaceTime. Once you have made your selections, click the “Apply” button
5. Click the “Select App” button (the FaceTime icon should still be highlighted)

The Apple Configurator blueprints have now been set up and can be loaded onto the iPads.

### Set Up iPads

This section will cover how to set up the patient and hub iPads using the AC2 blueprints created in the previous section. You will need to click buttons on both the iPad and the Mac running AC2 to complete these steps.

If your hospital has an Apple Device Enrollment Program account or a Mobile Device Manager, adjust the below steps as needed.

**Prepare iPads**

This step resets the iPad and claims it as part of your organization.

1. Plug in the iPad into the computer running Apple Configurator 2
2. On the iPad, click “Trust This Computer” on the popup window. You may need to enter your passcode to confirm.
3. In AC2, ensure you’ve exited from Blueprint editing mode by clicking the “Blueprints” icon > “Exit Blueprints,” which takes you to the “All Devices” screen. You should see the device that was plugged in.
4. Double-click on the iPad
5. Click the “Prepare” button in the top-middle of the screen. A popup menu will open.
   1. In the “Prepare Devices” menu, make the following choices, then click “Next”
      1. Manual Configuration
      2. Uncheck “Add to Device Enrollment Program”
      3. Check “Supervise Devices”
         1. Uncheck “Allow devices to pair with other computers”
   2. Choose “Do not enroll in MDM” and click “Next”
   3. Choose your organization and click “Next”
   4. On the “Configure iOS Setup Assistant” screen, make the following choices
      1. Setup Assistant: “Show only some steps”
      2. Uncheck everything except
         1. Passcode
         2. Apple ID
   5. Click “Prepare” to close this menu
      1. If you receive a popup that states “Configurator could not perform the requested action because “iPad” has already been prepared, to override this, click “Erase.”
6. The iPad will reboot. This may take a few minutes.
   1. If the iPad has previously been set up with a different Apple ID and hasn’t been factory reset before trying to prepare the iPad with AC2, you will see an error message in AC2 that says, “Unable to Activate,” but you will be able to continue setup. Do not click anything in the error message, and follow the steps below.
      1. Wait until the iPad finishes rebooting and proceed through the initial setup instructions on the iPad.
      2. After entering your WiFi credentials you will see a screen that says “Activation Lock”
      3. Enter the previous Apple ID and password for the iPad (the account the iPad was set up with previously). Do not continue through setup on the iPad.
      4. After entering the credentials from the above step, go back to AC2 and click “Try Again” on the error message that popped up
      5. Go back to step 5 and prepare the iPad again. It will work this time.
7. When you see the screen on the iPad with “Hello” in multiple languages, click the home button on the iPad to begin
8. Before proceeding, wait until the dialog box in AC2 disappears, indicating that the preparation is complete. On the iPad, you should see “Choose a Wi-Fi Network” and enter your WiFi information if not done via AC2.
9. Create a **Device Passcode**
10. Sign in with the Apple ID to use for this device
11. You will now be at the default iOS home screen with multiple apps visible. No blueprints have been applied yet.
12. Adjust the following two settings before applying the blueprint. After the blueprint has been applied for the patient iPad, the iPad will be in Single-App mode and settings will no longer be accessible.
    1. Open FaceTime. Using the volume buttons on the side of the iPad, increase the volume to the maximum volume.
    2. Go to Settings > Accessibility > Display & Text Size > Larger Text and choose a larger font size to help patients with decreased vision.
    3. Go to Settings > FaceTime and toggle off “FaceTime Live Photos” so that pictures can’t be taken while on a call

**Apply Blueprints**

1. In AC2, from the “All Devices” view, double click the iPad
2. In the icon bar at the top of the screen, click “Blueprints” and select the blueprint you want to apply to this iPad (patient vs hub)
3. Click “Apply” when prompted to confirm

If you use Family Sharing accounts for the patient iPads, the setup is almost complete. Using the hub iPad, edit the contacts for the entire “family.”

1. Open Contacts app and add each patient iPad that is in the same “family” as this hub
2. Open Settings app and select Screen Time (located on the left side menu)
3. Repeat the following steps until each patient iPad’s contacts have been set
   1. Under the “Family” section, click on the patient iPad’s name.
   2. Click “Communication Limits”
   3. Click “Contacts,” enter Screen Time passcode, click “Add New Contact,” and enter the Hub’s contact information to allow the patient iPad to call the Hub iPad.
   4. Click the back arrow at the top of the screen twice until you return to the menu listing the other patient iPad names

If you are not using Family Sharing for your patient iPad accounts, perform steps 10-12 from the “Manual Setup” section of this document (skip 11a, e, and g) to finish setup.

## Manual Setup

The process below is used to set up the iPads directly on each device and does not require a Mac for setup. The instructions assume the iPads are in a “factory reset” state. New iPads are in this state already. If using a donated or used iPad, first perform a factory reset. The current passwords for the iPad will be needed for the reset. Go to Settings > General > Reset > Erase all Content and Settings.

Assuming that the iPad is reset, the process for setup is:

1. Turn on the iPad
2. Follow the instructions until prompted for a 6-digit passcode
3. Generate a 6-digit passcode. Consider using a random number generator such as [random.org](https://www.random.org/integers/?num=100&min=1&max=999999&col=1&base=10&format=html&rnd=new). This number is your **Device Passcode**.
4. Follow the instructions, including setting up your WiFi, until prompted for an Apple ID
5. Enter one of the Apple IDs you created. Be sure to only use an Apple ID on one iPad; do not use the same Apple ID on multiple iPads.
6. Follow the instructions until you reach the home screen. Choose “no”, “don’t set up”, or “set up later” for most options as applicable
7. Once at initial setup is complete and the home screen shows, open “Settings” and perform the following settings changes below
   1. Display and Brightness > Auto Lock > Never
   2. Face ID and Passcode > Require Passcode > 4 hours or set another desired time interval
   3. General > Software Update > Download and Install
8. Wait for the software update to download and install
9. Once the iPad reboots after the software update, go through the options until you reach the home screen again
10. Open “Contacts” and add contacts as necessary
    1. For a “Patient” iPad, add the usernames for any “Hub” iPads you’d like the patient to be able to call
    2. For a “Hub” iPad, add the usernames for any “Patient” iPads you’d like the staff to be able to call
11. Open “Settings”
    1. Go to Apple Account (First item in “Settings” showing the account name)
       1. iCloud
          1. Turn off everything except for Contacts
          2. Ensure iCloud Backup is off
             1. This ensures that any data that winds up on the device won’t be stored in the cloud
             2. This also ensures that none of the data from the device can be decrypted
    2. Go to Sounds
       1. Set “Ringer and Alerts” to the maximum volume
       2. Turn off “Change with Buttons” so that the volume buttons on the side of the iPad change the audio volume rather than the ringer volume
    3. Go to Accessibility > Guided Access
       1. Turn on Guided Access
       2. Go to Passcode Settings > Set Guided Access Passcode
          1. Generate a 6-digit passcode. This number is your **Guided Access Passcode**.
    4. Go to Accessibility > Display & Text Size > Larger Text and choose a larger font size to help patients with decreased vision
    5. Go to Messages
       1. Turn off iMessage
          1. This is an additional security lock-down to ensure iMessage is not used under any circumstances on the iPad.
    6. Go to FaceTime
       1. Turn off FaceTime Live Photos
          1. This turns off FaceTime’s native screen capture tool so that pictures can’t be taken while on a call
    7. Go to Photos
       1. Turn off iCloud Photos
          1. Taking photos in FaceTime is disabled, but if an iPad is not properly restricted to FaceTime access only, this setting ensures any photos taken aren’t sent to the cloud
       2. Turn off Shared Albums
       3. Turn off Auto-play Videos and Live Photos
       4. Turn off Show Holiday Events
    8. Go to Screen Time
       1. This should automatically open a dialog box
          1. Mark that “this iPad is for a child”
          2. Click no/later for each question it asks you until you are prompted for a passcode
          3. Set a 4-digit **Screen Time Passcode**.
          4. Enter the Apple ID of the hub or system administrator’s account as the recovery ID (recovery ID cannot match the Apple ID of the current iPad for this setting)
             1. This will be the Apple ID used to attempt to reset the Screen Time passcode if it is lost
       2. Go to “Communication Limits”
          1. Go to “During Allowed Screen Time”
             1. Contacts Only
          2. Ensure “Allow editing of contacts” is off
       3. Go to “Always Allowed”
          1. Click on the red minus circle next to
             1. Maps
             2. Messages

Click OK in the dialog box that opens warning you not to do this

- - 1. Go to “Content and Privacy Restrictions”
       1. Turn on Content and Privacy Restrictions
       2. Go to iTunes and App Store Purchases
          1. Mark Don’t Allow for all
          2. Mark Always Require Password
    2. Go to “Allowed Apps”
       1. Turn off all except
          1. FaceTime
          2. Camera
    3. In the “Allow Changes” section at the bottom
       1. Mark all “Don’t Allow”

1. Open FaceTime. Using the volume buttons on the side of the iPad, increase the volume to the maximum volume. Once FaceTime is in Guided Access mode, users will not be able to change the volume, so this needs to be set ahead of time.
2. Go back to the home screen to set up the home screen
   1. Except for the apps below, group all other apps into a single folder
      1. Settings
      2. FaceTime
      3. Contacts
   2. Move the folder from (a) to the second page
   3. Move the 3 apps from (a) to the dock
   4. The home screen should now be empty except for the dock which should have the 3 apps

# iPad Preparation for Use

Prior to deploying the iPads for use in the hospital, the patient iPads need to be restricted to FaceTime only. If AC2 was used to prepare the patient iPads, no additional steps are necessary. If manual setup was used, open FaceTime and triple click the home button.
