# Supplementary material for: Telehealth Solutions for In-hospital Communication with Patients Under Isolation During COVID-19
Source: West J Emerg Med. 2020 Jun 23;21(4):801–6. doi: 10.5811/westjem.2020.5.48165 (PMC7390554; doi:10.5811/westjem.2020.5.48165)
Supplement: Supplementary file 3 [file wjem-21-801-s003.docx]

**Supplement 3.** iPad usage instructions.

[**Goal** 1](#_Toc39597979)

[**Introduction** 2](#_Toc39597980)

[Security 2](#_Toc39597981)

[Protocols 2](#_Toc39597982)

[**Patient Use Protocols** 2](#_Toc39597983)

[Option 1 - Communicate with Hospital Staff Only 2](#_Toc39597984)

[Overview 2](#_Toc39597985)

[Ratings 3](#_Toc39597986)

[Protocol 3](#_Toc39597987)

[Option 2 - Communicate with Staff and Loved Ones 4](#_Toc39597988)

[Overview 4](#_Toc39597989)

[Ratings 4](#_Toc39597990)

[Protocol 4](#_Toc39597991)

[Modifications 6](#_Toc39597992)

[Modification 1 - Longer Battery Life 6](#_Toc39597993)

[Overview 6](#_Toc39597994)

[Ratings 6](#_Toc39597995)

[Protocol Modifications 7](#_Toc39597996)

# **GOAL**

This document will cover the steps to use an iPad that has been set up using the instructions from Supplement 2 for in hospital telecommunication between patients and providers and between patients and families.

Based on use cases and which setup options were used (see Supplement 2), there are a few different variants for protocolizing iPad use for your hospital. Tradeoffs occur between ease of device management (user and staff burden) and iPad calling features. This document will cover the different variants and their advantages or disadvantages.

#

# **INTRODUCTION**

## **Security**

When set up fully as described in the iPad Setup document, it’s important to understand the security checks in place for any actions that the patient might be able to take with the iPads. Depending on the setup method used, not all passcodes may be needed.

There are four security measures in places on the devices:

1. Apple ID Password: protects Apple account that the iPad is logged into
2. Device Passcode: entered on lock screen to unlock iPad and for certain device settings
3. Screen Time Passcode: used to edit “parental control” restricted settings such as contacts
4. Guided Access Passcode: used to exit the one app that the iPad is locked into in Guided Access mode. In this case, it will be used to exit FaceTime

##

## **Protocols**

Based on the communication goals, here are a few protocol variations that can be adopted. This section describes a rating system for the different variants and optional modifications across the following categories:

1. Security
2. Ease of Use - Hospital Staff (non-IT staff)
3. Ease of Use - Patient
4. Ability for Patient to Call Family
5. Battery Life

Each protocol variant will receive a subjective rating between:

☆☆☆ (no stars) - Not Possible
★☆☆ (one star) - Not ideal
★★☆ (two stars) - OK
★★★ (three stars) - best

#

# **Patient Use Protocols**

## Option 1 - Communicate with Hospital Staff Only

### Overview

This protocol optimizes for iPad security and is the easiest for hospital staff. It minimizes steps required, but it does not give the ability for the patient to call their family.

In this protocol, the iPads are always on with the FaceTime app open, so no pass codes are needed. Check out an iPad from the storage location, plug it in, and hand it to the patient. Because the iPad is always on, we recommend using long charging cables to keep the iPad plugged in both while in use and during storage.

### Ratings

| **Category** | **Rating** | **Explanation** |
| --- | --- | --- |
| Security | ★★★ | Only calls to and from contacts allowed. No contacts are added except for the staff iPad. |
| Ease of Use - Staff | ★★★ | Easy to use; hand iPad to patient, no contacts to add or remove before use. No passcodes needed. |
| Ease of Use - Patient | ★★★ | Easy to use, single button to call the staff iPad |
| Able to Call Family | ☆☆☆ | Cannot call anyone except the staff iPad |
| Battery Life | ★☆☆ | iPad and screen always on, recommend plugging in at all times |

### **PROTOCOL**

**iPad Check Out**

1. Check out an iPad from the storage location. The iPad will be on and FaceTime will already be open.
2. Check that Wi-Fi is on. Verify that the Wi-Fi icon appears in the upper right corner of the screen. If not, proceed to the “Troubleshoot Wi-Fi” Section of the Additional Instructions
3. Give the patient the iPad and show the patient how to call providers.

**Patient Use of iPad**

1. Place/receive calls to/from staff only

**iPad Check In**

1. Clean iPad with hospital-approved disinfectant
2. Return iPad to storage location and plug in to charge

##

## **Option 2 - Communicate with Staff and Loved Ones**

### **Overview**

This option maintains security while also allowing patients to speak with their families/other contacts. Hospital staff caring for the patient will need to add the patient’s desired contacts to the iPad’s contact list before the patient can make or receive calls. Hospital staff must also remove contacts before returning the iPad so that the next patient does not receive calls from prior contacts. This option requires staff to have access to both the Guided Access and Screen Time passcodes.

### **Ratings**

| **Category** | **Stars** | **Explanation** |
| --- | --- | --- |
| Security | ★★★ | Only calls to and from contacts allowed. Contacts are added by hospital staff. Patients cannot add their own. |
| Ease of Use - Staff | ★★☆ | Staff need to manage contact lists and have 2 passcodes. Staff errors in contact lists creates a potential for patient privacy violations. |
| Ease of Use - Patient | ★★★ | Easy to use, 1-3 clicks to make a call |
| Able to Call Family | ★★☆ | Patients cannot freely make calls but can call loved ones who have been added to the contacts |
| Battery Life | ★☆☆ | iPad and screen always on, recommend plugging in at all times |

###

### **Protocol**

**iPad Check Out**

1. Check out an iPad from the storage location. The iPad will be on and FaceTime will already be open.
2. Check that Wi-Fi is on. Verify that the Wi-Fi icon appears in the upper right corner of the screen. If not, proceed to the “Troubleshoot Wi-Fi” Section of the Additional Instructions
3. Verification steps (verify iPad was reset appropriately when it was checked in). Verifying that the iPad was appropriately reset is an added level of security before allowing the next patient to use the iPad.
   1. Erase call log. For each non-hospital contact, swipe left and delete the call from the call log.
      *Calls made to/from the same contact appear as a single entry no matter how many times that contact was called, so the number of entries to be deleted depends on the number of outside contacts a patient called.*
   2. Remove non-hospital contacts.
      If manual setup (from Supplement 2) was used:
      1. Exit Guided Access mode by triple clicking the home button and entering the Guided Access passcode. Select “End” in the top left corner, and press the home button to exit FaceTime.
      2. Open the “Contacts” app and remove any non-hospital contacts. Click Edit in the upper right hand corner, enter Screen Time passcode, scroll to the bottom of the contact information, and click “Delete Contact”

If Apple Configurator (from Supplement 2) was used:

- - 1. Using the corresponding “parent”/staff iPad, open the “Settings” app. Click on “Screen Time” in the list on the left. Choose the appropriate patient iPad from the “Family” section, which will take you to the patient iPad’s Screen Time settings.
    2. Click “Communication Limits” and then “Contacts.” A new window with a list of contacts for that patient iPad will appear.
    3. For each non-hospital contact, click on the contact, click “Edit” in the upper-right corner, scroll to the bottom of the contact information, and click “Delete Contact”

1. Add the patient’s contacts. If the verification step was skipped, see item 3.2 above for how to access the patient iPad’s contacts.
   If manual setup (from Supplement 2) was used:
   1. Open the “Contacts” app. Click the “+” at the middle of the top of the screen. Enter Screen Time passcode. Enter contact details.
   2. Resecure the patient iPad. Click the home button. Open the FaceTime app. Triple click the home button to re-enable Guided Access mode so the only app that can be used is FaceTime.

If Apple Configurator (from Supplement 2) was used:

- 1. Access the patient iPad’s contacts according to steps 3.2.3 and 3.2.4.
  2. For each new contact, click on the “+” button in the upper right corner. Select “Add New Contact”. Enter the contact info, then click “Done” in the upper-right corner.

1. Give the patient the iPad and show the patient how to make calls.

**Patient Use of iPad**

1. Place/receive calls to/from staff and loved ones

**iPad Check In**

1. Clean iPad with hospital-approved disinfectant
2. Erase call log. For each non-hospital contact, swipe left and delete the call from the call log.
   *Calls made to/from the same contact appear as a single entry no matter how many times that contact was called, so the number of entries to be deleted depends on the number of outside contacts a patient called.*
3. Remove non-hospital contacts.
   If manual setup (from Supplement 2) was used:
   1. Exit Guided Access mode by triple clicking the home button and entering the Guided Access passcode. Select “End” in the top left corner, and press the home button to exit FaceTime.
   2. Open the “Contacts” app and remove any non-hospital contacts. Click Edit in the upper right hand corner, enter Screen Time passcode, scroll to the bottom of the contact information, and click “Delete Contact”
   3. Resecure the patient iPad. Click the home button. Open the FaceTime app. Triple click the home button to re-enable Guided Access mode so the only app that can be used is FaceTime.

If Apple Configurator (from Supplement 2) was used:

- 1. Using the corresponding “parent”/staff iPad, open the “Settings” app. Click on “Screen Time” in the list on the left. Choose the appropriate patient iPad from the “Family” section, which will take you to the patient iPad’s Screen Time settings.
  2. Click “Communication Limits” and then “Contacts.” A new window with a list of contacts for that patient iPad will appear.
  3. For each non-hospital contact, click on the contact, click “Edit” in the upper-right corner, scroll to the bottom of the contact information, and click “Delete Contact”

1. Return iPad to storage location and plug in to charge

##

## **Modifications**

This section will cover modifications that can be applied to the base protocol options. The ratings charts in this section will show how ratings change as a result of the modifications.

### Modification 1 - Longer Battery Life

####

#### **Overview**

This modification adds a couple extra steps for the staff when they check out and check in the iPads, but the iPads will not have to stay on while not in use. This change will extend iPad’s battery life and will potentially extend overall device life as the iPad can get warm when it is always on. Staff will need access to the device passcode to use this modification, which requires staff to know 2-3 passcodes depending which “Options” protocol was selected.

####

#### **Ratings**

| **Category** | **Stars** | **Explanation** |
| --- | --- | --- |
| Security | No change | No change |
| Ease of Use - Staff | -1 ☆ | Staff now need to turn on/off iPads before/after use and will need access to 2-3 passcodes. |
| Ease of Use - Patient | No change | No change |
| Able to Call Family | No change | No change |
| Battery Life | +1 ★ | iPad now off when not in use |

####

#### **Protocol Modifications**

**iPad Check Out**

Before doing the checkout procedure described in the “Options” sections:

1. Turn on the iPad by holding the lock button down until the screen turns on.
2. Enter the device passcode
3. Open the Facetime app if not already open
4. If manual setup (from Supplement 2) was used:
   Enable Guided Access mode by triple clicking the home button.

**Patient Use of iPad**

No change

**iPad Check In**

After doing the check in procedure described in the “Options” sections:

1. Exit Guided Access mode by triple clicking the home button and entering the Guided Access passcode. Select “End” in the top left corner.
2. Turn off iPad by holding down the lock button until a slider appears on the screen. Slide the slider and the iPad will turn off.
